# The Relationship between Syntactic Satiation and Syntactic Priming: A First Look

**DOI:** 10.3389/fpsyg.2017.01851

**Published:** 2017-10-25

**Authors:** Monica L. Do, Elsi Kaiser

**Affiliations:** Department of Linguistics, University of Southern California, Los Angeles, CA, United States

**Keywords:** satiation, syntactic priming, island effects, processing difficulty, experimental syntax, acceptability judgments

## Abstract

Syntactic satiation is the phenomenon where some sentences that initially seem ungrammatical appear more acceptable after repeated exposures (Snyder, 2000). We investigated satiation by manipulating two factors known to affect syntactic priming, a phenomenon where recent exposure to a grammatical structure facilitates subsequent processing of that structure (Bock, 1986). Specifically, we manipulated (i) *Proximity of exposure* (number of sentences between primes and targets) and (ii) *Lexical repetition* (type of phrase repeated across primes and targets). Experiment 1 investigated whether acceptability ratings of Complex-NP Constraint (CNPC) and Subject islands improve as consequence of these variables. If so, priming and satiation may be linked. When primes were separated from targets by one sentence, CNPC islands’ acceptability was improved by a preceding island of the same type, but Subject islands’ acceptability was not. When prime-target pairs were separated by five sentences, we found no improvement for either island type. Experiment 2 asked whether improvements in Experiment 1 reflected online processing or offline end-of-sentence effects. We used a self-paced reading paradigm to diagnose online structure-building and processing facilitation (Ivanova et al., 2012a) during processing. We found priming for Subject islands when primes and targets were close together, but not when they were further apart. No effects were detected when CNPC islands were close together, but there was a localized effect when sentences were further apart. The disjunction between Experiments 1 and 2 suggests repetition of the structure in Subject islands facilitated online processing but did not ‘spill over’ to acceptability ratings. Meanwhile, results for CNPC islands suggest that acceptability rating improvements in Experiment 1 may be driven by factors distinct from online processing facilitation. Together, our experiments show that satiation may not be a one-size-fit-all phenomenon but, instead, appears to manifest itself differently for different types of structures. Priming is possible and may be linked to satiation in *some* purportedly “unbuildable” structures (e.g., Subject islands), but not for all types (e.g., CNPC islands). Despite this, it appears that while the types of mechanisms targeting different island types are distinct, they are nevertheless similarly sensitive to the proximity between individual exposures.

## Introduction

Syntactic satiation is the phenomenon where some sentences that are “initially judged ungrammatical begin to sound increasingly acceptable” after repeated exposures (Snyder, 2000, p. 575). Anecdotally, this phenomenon is not new; most linguists have, at one time or another, fallen victim to “linguists’ disease.” Experimentally, though, evidence for satiation has yielded mixed results. So, while prior work has laid the groundwork for investigation, a number of fundamental questions remain – including the issue of which structures can/cannot satiate. Consequently, answering the subsequent questions of what mechanism and what factors underlie satiation has been challenging.

Existing work suggests that *only certain* syntactic violations satiate, while others are consistently perceived as unacceptable despite repeated exposure (e.g., Snyder, 2000; Sprouse, 2009). These structural asymmetries show that this poorly understood phenomenon has far-reaching implications for linguistic methodology (e.g., the design of acceptability judgment studies), for linguistic theories (e.g., the relative strength and status of syntactic violations, etc.), and for language processing theories (e.g., how the processor mentally represents ungrammatical sentences).

The present work investigates syntactic satiation from a new methodological and theoretical angle by manipulating variables known to affect a similar – though comparatively well-attested and better-understood phenomenon – known as syntactic priming (a.k.a. structural priming). Specifically, syntactic priming is a phenomenon where recent exposure to a given structure facilitates subsequent processing of that same structure (Bock, 1986; see Branigan, 2007 for a review). For instance, if a speaker has been recently exposed to a passive sentence (e.g., ‘The cat was chased by the dog’; the prime), she is more likely to produce another passive sentence (the target) the next time she is faced with a choice between an active and a passive structure (e.g., Bock, 1986).

The two phenomena of priming and satiation appear to resemble each other: In both cases, it’s *exposure* that influences how structures are processed. Despite this similarity, though, the literatures on priming and satiation have developed in relative isolation from one another. This may be partly due to differences in their methodological traditions. Priming, for instance, has been investigated almost exclusively with grammatical sentences (but see Kaschak and Glenberg, 2004; Ivanova et al., 2012a,b, 2017; etc.), often by means of production-oriented methods where the dependent variable is the proportion of trials on which a participant produces the primed structure. There have also been comprehension-oriented studies of priming (see Tooley and Traxler, 2010 for review), where the dependent variable is often ease of processing (as measured by eye-tracking, ERP, self-paced reading, etc.). Satiation, by contrast, has used offline acceptability judgments to see whether increased exposure improves the acceptability of ungrammatical sentences. Prior work on satiation has not made any direct claims about ease of processing for these ungrammatical sentences. Consequently, the broader relationship between priming and satiation has been one of ‘apples and oranges’ as the potential relationship between these two phenomena has largely been overlooked.

Our work makes a first attempt at bridging these fields by using a priming-style design to investigate the mechanisms that may underlie satiation in two structures said to be ungrammatical in English, Complex Noun-Phrase Constraint (CNPC) islands and Subject islands. We present two experiments which approach satiation in a new way by manipulating two factors – namely (a) the proximity of prime and target sentences, and (b) the type of lexical repetition that occurs between them – known to affect syntactic priming.

Experiment 1 applies those factors to an offline acceptability rating task to test for rating improvements in CNPC and Subject islands. Acceptability ratings showed that CNPC islands were improved by a preceding CNPC structure. Subject islands, by contrast, did not appear to be affected by our manipulations. Moreover, improvements in CNPC islands occurred when primes and targets were separated by one intervening sentence, but not when sentences were separated by five interveners. Experiment 1 results suggest that priming may be linked to satiation, but that its effects may be dependent on the type of syntactic structure and the proximity of exposure between prime and target sentences.

Experiment 2 used word-by-word self-paced reading times to investigate whether acceptability rating improvements from Experiment 1 corresponded to processing facilitation during moment-by-moment comprehension. However, we first conducted a stop-being-grammatical-task, in order to (i) address potential concerns regarding the point at which readers perceive CNPC islands and Subject islands as being ungrammatical, and to (ii) guide the interpretation of the self-paced reading results in Experiment 2. In Experiment 2, in contrast to the offline acceptability ratings, *online* reading time measures detected priming in Subject islands: Reading times for Subject islands were faster when participants had just seen another Subject island, but only when primes and targets were close together. Surprisingly, despite offline rating improvements, we found no priming (no reading time facilitation) for CNPC islands in Experiment 2 when primes and targets were close together. We observed a priming effect localized to one word when CNPC islands were separated by five sentences.

Together, our results suggest that satiation may be a more nuanced phenomenon than previously thought: It appears to be dependent on the type of structure under investigation and its observability depends on the method used to investigate it. Consistent differences between CNPC and Subject islands in Experiments 1 and 2 lead us to believe that what has been viewed as a unified phenomenon of ‘satiation’ in both CNPC and Subject islands may not be unified after all: We may be dealing with two different phenomena that are only be superficially similar. Based on our results, we suggest that different mechanisms may be at work during the processing of CNPC and Subject islands. Our results also suggest that the proximity between individual exposures plays a role in both the offline acceptability and online comprehension of these island types.

### Syntactic Satiation

Work in syntactic satiation has typically focused on ‘island’ structures (ex. 3–4), *wh-*questions which are ungrammatical in English because they are said to violate constraints governing the movement of *wh-*phrases in English.

(1) Who does Mary believe that John likes ______ ?
(2) What does John know ____ fell on the floor?
(3) ^∗^Who does Mary believe [the claim] that John likes ____? [CNPC Island]
(4) ^∗^What does John know (that) [a bottle of ____] fell on the floor? [Subject Island]

More specifically, well-formed English questions (ex. 1–2) involve the creation of a ‘filler-gap dependency’ between the pronounced (the filler) and interpreted (the gap) *wh-*phrases. Though this dependency can span across multiple clauses, there are nevertheless conditions that govern the formation of the filler-gap dependency. When these conditions are violated, movement of the *wh-*filler to the front of the sentence is disallowed. In example (3), for instance, introducing a noun phrase (‘the claim’) between the filler and the gap embeds the *wh-*gap within a noun phrase from which *wh-*movement is not possible. Likewise, when the *wh-*gap appears within a subject phrase (‘a bottle of’), as in (4), the resulting sentence is ungrammatical. Because these phrases – namely, complex noun phrases and subjects, respectively – block the formation of *wh-*dependencies, they are considered ‘islands’ to extraction (here represented using brackets).

In the first experimental investigation of satiation, Snyder (2000) asked native English speakers to rate the grammaticality of several types of island structures.^1^ Participants rated each sentence type a total of five times. To determine whether there had been any improvement in ratings, the number of ‘grammatical/acceptable’ responses in the first two vs. the last two exposures was compared. Sentences were said to improve, or ‘satiate,’ if there were more ‘grammatical/acceptable’ responses in the second half than in the first half of the study.

Notably, Snyder (2000) found that while some ungrammatical structures satiated, others did not.^2^ However, more recent work has been unable to replicate some of these original findings. For instance, the satiation effects initially observed for CNPC islands have been replicated by some (e.g., Sag et al., 2007; Hofmeister and Sag, 2010; Goodall, 2011; Snyder, 2017 using acceptability ratings), but not by others (Hiramatsu, 2000 using Likert scale ratings; Sprouse, 2009 using magnitude estimation). In addition, related work by Sag et al. (2007) and Hofmeister and Sag (2010) investigated CNPC islands using self-paced reading where participants were asked to read two types of CNPC islands word-by-word: In the first type, *wh-*fillers were bare *wh-*phrases (e.g., ‘who’ or ‘what’), whereas in the second type, the *wh-*fillers were more informative *which*-NP phrases (e.g., ‘which convict’), which have been shown to be more acceptable (Karttunen, 1977; Maling and Zaenen, 1982; Pesetsky, 1987, 2000; etc.). Both Sag et al. (2007) and Hofmeister and Sag (2010) reported a similar result. Participants rated *which*-NP CNPC islands more acceptable than CNPC islands with bare *wh-*phrases. Additionally, reading times for CNPC islands with *which*-NPs did not differ from their grammatical, non-island counterparts. Results from both these studies were taken as evidence that under some circumstances, processing costs for CNPC islands could be drastically attenuated strictly by manipulating a single *processing*-related factor [(namely, the informativeness of the *wh-*element; but see Goodall (2015) for evidence of residual island effects even with highly informative filler phrases)]. We return to this point in the discussion.

Subject islands have been under similar debate. Although Snyder (2000) only showed a marginally significant effect of satiation, Hiramatsu (2000), Francom (2009), and Chaves and Dery (2014) have found significant satiation effects for Subject islands. Work by others, however, either replicated Snyder’s (2000) marginal effects (e.g., Snyder, 2017) or failed to detect satiation effects in these island types (e.g., Sprouse, 2009; Goodall, 2011; Crawford, 2012; etc.).

In sum, at issue is not only the question of (i) what mechanisms underlie satiation, but also the more fundamental question of (ii) whether what has been termed ‘satiation’ in CNPC and Subject islands is even the same phenomenon. In part because the basic facts of satiation remain unclear (e.g., there is no consensus regarding which structures do and do not satiate), it has been difficult to interpret what satiation as a phenomenon means both for experimental and for theoretical linguistics.

At a minimum, investigations into the phenomenon of satiation represent a methodological question for the design of acceptability judgment studies. For instance, a better understanding of the factors underlying satiation may have consequences for understanding individual variation in judgments, the number of times target items may be repeated, proximity of individual target items to each other, etc. Beyond that, satiation potentially implicates the interaction between grammatical constraints and how those constraints are mentally represented. This is particularly true in the case of grammatical violations, like CNPC and Subject islands, whose status in both the experimental and theoretical literature is still under debate.

### Syntactic Priming

Unlike satiation, syntactic priming – where exposure to a syntactic structure can facilitate subsequent processing of that same structure (Bock, 1986) – is a well-known and well-attested phenomenon. A large body of work (e.g., Bock, 1986; Branigan et al., 1995; Pickering and Branigan, 1998; Bock and Griffin, 2000) in priming has shown that speakers are better able to access structures (e.g., passive sentences) that they’ve previously been exposed to. And, though most of the research in priming focuses on production, similar priming effects have also been found in studies of comprehension. In general, the ability to facilitate access to recently exposed structures has been attributed to two complementary mechanisms that are not mutually exclusive (Hartsuiker et al., 2008): (1) residual activation of combinatorial nodes in a syntactic structure (often lexically based), resulting in a short-lived priming effect (e.g., Pickering and Branigan, 1998; Branigan et al., 1999) and (2) Implicit learning of mappings between message-level representations and syntactic structures, resulting in a longer-term priming effect (Bock and Griffin, 2000; Chang et al., 2006; *inter alia*).

Residual activation accounts typically locate priming in the lexical units which connect to the larger syntactic structure (e.g., Pickering and Branigan, 1998; Branigan et al., 1999; Pickering et al., 2000; though see Scheepers, 2003). Since recent exposure momentarily increases the activation level of syntactic structures, priming occurs when the parser selects structures which are more active in memory, e.g., structures with higher residual activation levels. Because these accounts attribute priming to the moment-by-moment activation levels of particular lexicon-to-structure combinations, they also predict a short-term time course for priming (e.g., Roelofs, 1992; Pickering and Branigan, 1998). In particular, because the activation of lexical units is believed to decay quickly and automatically, priming effects are short-lived. Further, because residual activation accounts take priming to involve the links between lexical units and their larger syntactic structure, this account also predicts a stronger priming effect when prime and target sentences share lexical items (e.g., Pickering and Branigan, 1998; Cleland and Pickering, 2003). Indeed, this ‘lexical boost’ effect has been replicated in a number of production studies (e.g., Pickering and Branigan, 1998; Cleland and Pickering, 2003; Bernolet et al., 2013) and in nearly all comprehension studies (see Tooley and Traxler, 2010 for review).^3^ But, other work has shown that priming can still occur absent lexical repetition in production (e.g., Pickering and Branigan, 1998; Scheepers, 2003; Kaschak and Glenberg, 2004; Hartsuiker et al., 2004) and comprehension (e.g., Luka and Barsalou, 2005; Thothathiri and Snedeker, 2008a,b; Traxler, 2008; Ivanova et al., 2012a,b).

A second mechanism contributing to structural priming – implicit learning – attributes priming to changes that occur independent of the lexicon; so, lexical repetition between prime and target sentences is not predicted to influence the strength of priming (Bock and Griffin, 2000; Chang et al., 2000, 2006; Bock et al., 2007). Rather, priming occurs as the result of cumulative, lasting learning from experience: Encountering a given message with a given structure reinforces learning of that meaning-to-message mapping. Consequently, the structure becomes more accessible the next time the processing system encounters the same type of message. Because priming under this account is the by-product of cumulative changes at the abstract structural level, priming is predicted to be relatively long-lasting (e.g., Hartsuiker and Kolk, 1998; Bock and Griffin, 2000; Bock et al., 2007; Hartsuiker et al., 2008). Work by Bock and Griffin (2000) measured the proportion of prepositional datives that participants produced after hearing a prepositional dative prime (e.g., “A boy is giving an apple to a teacher.”) or a double-object prime (e.g., “A boy is giving a teacher an apple.”). To test the longevity of priming, they varied the number of unrelated sentences intervening between the prime and target structures. Consistent with prior work hinting at the persistence of priming, they found that effects could persist through as many as 10 intervening sentences.

The role of *ungrammatical* structures, though, is unclear. Most work in priming has focused on structural facilitation in the context of fully grammatical sentences – sentences whose structures *can* be mentally represented by the comprehender. Some researchers argue against the possibility of priming in ungrammatical sentences. For example, Sprouse (2007) suggests that priming “is predicated upon the existence of a licit representation. Given that ungrammatical structures have no licit representation… there should be no syntactic priming effect for ungrammatical structures” (Sprouse, 2007, p. 128). In contrast, other work (Kaschak and Glenberg, 2004; Luka and Barsalou, 2005; Ivanova et al., 2012a,b, 2017; etc.) has suggested that priming need not be limited to fully grammatical sentences.

At the lexical level, a series of experiments by Ivanova et al. (2012a,b, 2017) investigated if and how comprehenders build syntactic representations for anomalous ditransitive sentences (ex. 5a–b), when the verb is (a) a nonce word void of any semantic meaning, (b) a grammatically unacceptable verb, or (c) missing altogether. These anomalous sentences were compared against a fully grammatical counterpart (d).

(5a) The waitress brunks the book to the monk/The waitress brunks the monk the book.
(5b) The waitress exists the book to the monk/The waitress exists the monk the book.
(5c) The waitress the book to the monk/The waitress the monk the book.
(5d) The waitress gave the book to the monk/The waitress gave the monk the book.

Crucially, Ivanova et al. (2012a, 2017) used the presence/absence of syntactic priming effects (assessed via the proportion of participant-produced sentences matching the structure of the prime) to diagnose whether comprehenders had built syntactic representations for anomalous sentences.^4^ They found evidence of structural priming – and thus the presence of abstract syntactic structure – with nonce-verb primes (5a), with illicit verb primes (5b) and even when the prime contained no verb (5c). Thus, work by Ivanova et al. (2012a, 2017) suggests that even when comprehenders encounter incomplete and/or ungrammatical sentences, they do not “abandon” the syntactic route altogether. In addition to using other available information, comprehenders do attempt to construct a representation for the sentence via syntax.

An open question, though, is whether findings from Ivanova et al. (2012a,b, 2017) can be straight-forwardly extended to account for structures as degraded as island structures (ex. 3–4). Anomalies in those works were largely localized to a single, albeit structurally important, lexical item – namely, the verb. Indeed, Ivanova et al. (2012b) themselves raise the question of whether their results may generalize to sentences where the locus of ungrammaticality extends beyond the level of individual lexical items – e.g., as in island structures (Ivanova et al., 2012b, p. 367).

Earlier work by Kaschak and Glenberg (2004) and Luka and Barsalou (2005) provide insights into what happens on the sentence level, although they did not test island structures. Specifically, Kaschak and Glenberg (2004) found priming-like effects in structures like ‘These vegetables need cooked.’, which are acceptable in some dialects, but ungrammatical in standard American English. In their experiment, half of the participants were exposed to the ‘needs’ structure during an initial training phase while the other half did not undergo training. Afterward, all participants were asked to read structurally similar sentences, such as ‘The valiant hero wants recognized for his courageous actions.’ Kaschak and Glenberg (2004) found faster word-by-word reading times for the novel ‘wants’ structures only for participants who had participated in the training session. This, they argued, provided evidence that participants were “learning to comprehend” the novel structure via a new meaning-to-message mapping (e.g., through implicit learning). Similar work by Luka and Barsalou (2005) investigated priming in a variety of moderately ungrammatical structures (e.g., ‘I miss having any time to do anything.’, ‘Who did you hire because he said would work hard?’). Participants first read sentences that were structurally similar to the target sentences, and after a 5-min break, rate the acceptability of the target sentences. Luka and Barsalou (2005) found acceptability improvements in as little as one prior exposure to a structurally similar sentence.

Taken together, these results indicate that priming may, indeed, be possible even with structures that *initially* seem unacceptable. Nevertheless, because work examining priming with ungrammatical sentences is relatively new, the limits of this priming effect are still unclear and the mechanisms and/or processes that underlie priming in ungrammatical sentences are not yet well-understood. Moreover, prior work has tended to either look at only one specific kind of anomaly, or has grouped together various types of ungrammatical sentences without comparing them systematically. Thus, it is not yet known how generalizable prior findings are, or whether different kinds of ungrammaticality may pattern differently with regard to the possibility of priming.

### The Current Study

The current work uses methods established in priming research to guide investigations into satiation, and in so doing, aims to shed light on broader issues related to the representation of ungrammatical sentences. Given the parallels between syntactic satiation and syntactic priming – namely, that both are linked to increased exposure – it may be possible for the underlying mechanism(s) responsible for satiation to be related to those in priming. The current work aims to contribute to our understanding of satiation and priming in three ways:

(1) Traditional approaches to satiation compared acceptability judgments over the course of an entire experiment, looking at cumulative effects on a ‘global’ level. By contrast, we test for improvements between prime and target pairs – ‘local,’ exposure-by-exposure comparisons – to see how single exposures to an ungrammatical prime can influence the acceptability of the subsequent target. Given that satiation effects have been notoriously difficult to replicate, even when studies have used similar materials, similar methods, and/or similar analyses (see *Syntactic Satiation*), looking at satiation through the lens of priming may provide independent evidence for how to interpret the facts of satiation.
(2) Whether structure-building is possible at all for ungrammatical, potentially ‘unrepresentable’ sentences like CNPC and Subject islands is an open question. Following Ivanova et al. (2012a,b, 2017), we use the presence of syntactic priming as a diagnostic for syntactic representation-building in cases where the input may be extremely degraded. In doing so, we examine not only the limits of representation-building, but also the ability of the processor to adapt to highly degraded input. Thus, our results also have implications for our understanding of the mental representations that underlie syntax, especially in the context of structures that may not be fully represented/representable in comprehenders’ minds.
(3) Finally, if comprehenders do, indeed, build syntactic representation of ungrammatical island sentences, an open question is to what extent processing of those representations may be similar to processing *grammatical* representations. We therefore “import” factors known to affect priming into our investigation of satiation to investigate the comparability of these two phenomena.

## Experiment 1: Acceptability Ratings

If proximity of exposure and lexical repetition – two factors known to modulate priming effects – can also increase the acceptability of CNPC and Subject islands, this might provide initial evidence that the same mechanisms underlying processing of grammatical sentences may play a role in how comprehenders’ evaluate notions of “(un)acceptability.” In other words, given that satiation is traditionally defined as increased acceptability, testing whether offline measures are influenced by processing-related factors is a key first step in determining whether priming and satiation are related.

Prior work in priming has shown that altering the number of sentences intervening between a prime and target can provide some insight into the mechanisms that contribute to priming. Because residual activation of a syntactic representation is short-lived, priming via this mechanism occurs when prime-target pairs are proximate, but not when they are further apart. By contrast, priming as an implicit learning effect appears to be long-lived (see *Syntactic Priming*). Thus, manipulating the *proximity* between prime and target sentences can shed light on one aspect of the underlying mechanism for satiation. We operationalize this by changing the number of sentences (either one unrelated sentence, referred to as Lag1, or five unrelated sentences, referred to as Lag5) that intervene between a prime (the initial exposure sentence) and its target (the subsequent test sentence). Additionally, residual activation and implicit learning accounts with respect to the presence of a ‘lexical boost’ when primes and targets share lexical items critical to the syntactic structure (e.g., phrase heads, see *Syntactic Priming*). Therefore, we also manipulate *lexical repetition* between prime and target sentences by comparing repetition of a phrase crucial to the island-forming structure vs. repetition of lexical items unrelated to the island itself.

### Materials and Methods

#### Participants

Eighty-four adult American English speakers, recruited via Amazon Mechanical Turk and paid $2 (Lag1 group) or $3 (Lag5 group), were included in the final analyses (*n*_Lag1_ = 40, *n*_Lag5_ = 44^5^).

#### Procedure

Participants saw sentences one at a time and rated how “natural or unnatural” each sentence “intuitively” sounded to them using a scale of 1 = “Completely Unacceptable” to 5 = “Completely Acceptable.” They were asked to rate sentences without reference to previously seen items and backtracking was disabled. The study was conducted using Qualtrics^6^ (version 2015; Qualtrics, Provo, UT).

#### Design

The number of sentences separating each prime from its subsequent target was varied between subjects: Prime-target pairs were separated either by one unrelated sentence (Lag1) or by five unrelated sentences (Lag5). Crucially, the total number of prime-target pairs was the same across both Lag1 and Lag5 versions; only the number of sentences intervening between primes and their targets varied. Specifically, participants rated three sets of prime-target pairs per condition (**Table 1**), for a total of six pairs in each sentence type and 12 prime-target pairs altogether. Additionally, participants rated 54 or 126 filler/intervener sentences in Lag1 and Lag5, respectively; these did not include island-related violations. Moreover, to address concerns that participants might “give up on” or adopt a response equalization strategy (Sprouse, 2009), participants rated a roughly equal number of ungrammatical and grammatical sentences over the course of entire study.

**Table 1:** Sample sentences (primes and targets) used in Experiments 1 and 2.

Sentence type	Repetition type	Trial type	Example sentences
CNPC	Island	Prime	Who did Richard dispute the claim that the paparazzi stalked?
		Target	Who did John deny the claim that the princess married?
	Unrelated	Prime	Who did Richard **deny** the allegation that the paparazzi stalked?
		Target	Who did John **deny** the claim that the princess married?
Subject	Island	Prime	What did opponents of hang a giant banner at the capitol?
		Target	What did opponents of start a violent riot outside the mall?
	Unrelated	Prime	What did fans of hang a giant banner **outside the mall?**
		Target	What did opponents of start a violent riot **outside the mall?**


#### Sentence Types

Based on prior work (Kluender and Kutas, 1993; Phillips, 2006; Sag et al., 2007; Hofmeister and Sag, 2010; etc.), we expect processing difficulty (gauged via reading time slowdowns) to arise at word 5 for CNPC and Subject islands (see **Table 2**), but crucially, for different reasons. In both cases, the parser begins actively searching for a *wh-*gap as soon as it encounters the sentence-initial *wh-*phrase (‘Who’/‘What’; Crain and Fodor, 1985; Frazier and Clifton, 1989; Gibson and Hickok, 1993; etc.). In CNPC islands, the processing difficulty expected at word 5 can be attributed to what is known as the filled-gap effect: The parser posits a gap for the *wh-*filler at the first possible position, word 5 (**Table 2**); but, when it encounters the head of the island phrase (‘the’) here, the parser realizes that this is not a possible position for the *wh-*gap and must revise its initial parse. We also expect a secondary site of processing difficulty at word 7, where the parser encounters the complementizer (e.g., ‘that’). Here, because the complementizer signals the end of the previous clause and because there was no available gap position in the initial clause, the parser should recognize that the *wh-*filler has been extracted from within an embedded clause headed by a complex-NP – in other words, that the *wh-*filler has been extracted from within a CNPC island. Thus, the expected processing difficulty at word 7 would correspond to the point where the parser has recognized the illicit, ungrammatical extraction. Indeed, these predictions are in line with what we observe in the stop-being-grammatical task (see *Differences between CNPC and Subject Islands: The Stop Being Grammatical Task*): Some comprehenders *begin* perceiving CNPC islands as unacceptable at word 5 with the majority of comprehenders judging CNPC islands to be unacceptable by word 7.

**Table 2:** Sample Complex-NP Constraint (CNPC) and Subject island sentences with corresponding word numbers. Shaded region denotes region of interest.

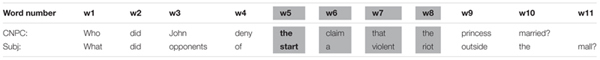

For each sentence type, targets were held constant but prime sentences were manipulated such that primes and targets either lexically repeated (i) the island-forming DP blocking *wh-*extraction or (ii) a phrase unrelated to the island (the matrix verb in CNPC islands and adjunct expressions in Subject islands). These four repetition conditions (**Table 1**) were varied within subjects and rotated using a standard Latin Square design. (Note that repetition types are not compared to a no-repetition baseline).

Finally, in order to prevent the possibility that a ‘target’ could also function as a ‘prime’ for subsequent sentences, individual pairs of primes and targets were separated by at least 10 unrelated sentences. Comprehension questions were also interspersed throughout the experiment to further increase the distance between pairs of primes and targets (and to ensure people paid attention).

We now make several notes regarding the construction of our materials. First, complex-NP phrases can sometimes be reanalyzed as a single constituent (e.g., “make the claim” can be reanalyzed as “claim”). In cases of reanalysis, these ungrammatical sentences become fully grammatical because the *wh-*filler is no longer extracted from within a CNPC island (Cinque, 1990; Davies and Dubinsky, 2003; etc.). To minimize the possibility of reanalysis, we chose TP-complements to the VP that did not seem easily reducible to a single VP. Additionally, work by Phillips (2006) has shown that positing a gap inside of Subject islands (parasitic gaps) is not only possible inside island structures but can also “rescue” otherwise ungrammatical sentences. However, as noted by Phillips, parasitic gapping may be limited to infinitivals, so we test only finite clauses where “gap creation [is] not attempted” (Phillips, 2006, p. 813). Finally, given that prior work has shown satiation even with bare *wh-*phrases (Chaves and Dery, 2014), we use only bare *wh-*phrases to avoid additional processing confounds associated with more informative *wh-*fillers (Sag et al., 2007; Hofmeister and Sag, 2010).

### Predictions

If the same factors known to influence priming – namely, the proximity between individual (prime-to-target) exposures and the type of lexical overlap between structures – produce higher acceptability ratings for target sentences than for primes, this suggests that acceptability ratings may be sensitive to the same factors that affect processing. Such a finding would provide reason to suspect that priming and satiation can be linked to the same underlying mechanisms. Alternatively, if we observe no rating improvements between primes and targets, this would not rule out the possibility of a relationship between satiation and priming, but would make any such relationship indirect.

In priming, the proximity of exposure between prime-target pairs has been used to distinguish between effects arising from short-term residual activation decay and/or longer-term effects arising from implicit learning. We use this same logic to investigate whether rating improvements (satiation) may be short- or long-term. If acceptability ratings from prime to target sentences improve (i.e., satiate) when primes and targets are close together (Lag1; one intervening sentence), but show small improvements or no improvements when they are far apart (Lag5: five intervening sentences), this may point to satiation being a short-lived effect that decays over time. But, if both lags show comparable rating improvements, this could point to satiation as a long-term effect analogous to implicit structure-learning.

Finally, lexical repetition often elicits a (short-lived) strengthening of the priming effect. According to residual activation accounts, this is because lexical repetition facilitates access to previously built syntactic structures. If acceptability is also sensitive to lexical repetition, we might find an analogous acceptability-rating ‘boost’ in Lag1 (primes and targets are close together) when prime-target pairs share lexical items. In particular, we may see stronger effects when the head of the syntactic island is repeated – given the significance of the head noun in the island structure – than when phrases unrelated to the island are repeated.

### Results

#### Data Analysis

We measured changes in acceptability ratings (on a five-point scale) from prime to target sentences in CNPC and Subject islands. All statistical analyses were performed on *z*-scores computed from each participants’ mean response to all experimental items. This helped control for differences in how individual participants would approach the five-point scale. However, analyses over raw ratings showed the same basic pattern of results. For ease of visual interpretation, graphs show raw ratings, not *z*-scores.

Statistical analyses were done in R (version 3.3.2; R Core Team, 2016) using linear mixed-effects regression models from the lme4 package (Bates et al., 2015). The Lag1 and Lag5 groups were compared independently. In all analyses, we included Sentence type (CNPC or Subject islands), Repetition Type (head of the island or an unrelated phrase), and Trial Type (prime vs. target sentence) as well as their interactions as fixed effects. We also incorporated by-subjects and by-items adjustments to the slopes and intercepts, which were reduced using model comparison.^7^ Effects were judged to be significant if |t|≥ 2.

#### Acceptability Ratings for Lag1

**FIGURE 1:**
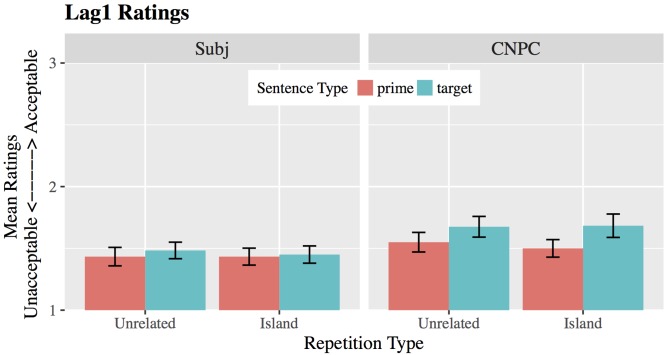
Mean ratings for Complex-NP Constraint (CNPC) and Subject islands in Lag1. Raw scores are presented on a 5-point scale, where 1 = Completely Unacceptable and 5 = Completely Acceptable. Error bars represent ±1 standard error. For visibility, we show only 1–3 points on the scale.

Mean ratings for sentences in the Lag1 group are shown in **Figure 1**. Overall, CNPC islands were rated significantly higher than Subject islands (b = 0.09, *SE* = 0.03, |t| = 2.82). Moreover, ratings for CNPC target sentences were higher than for primes regardless of repetition type. By contrast, ratings for prime and target Subject island sentences do not differ.

Statistically, there was a significant effect of trial type (β = 0.05, *SE* = 0.02, |t| = 2.3), but this was modulated by a marginal sentence-by-trial interaction (β = 0.09, *SE* = 0.05, |t| = 1.81). The presence of the interaction effect suggests that priming does not occur across the board: Target sentences were more acceptable than primes in CNPC islands (β = 0.1, *SE* = 0.04, |t| = 2.67), but not Subject islands (β = 0.01, *SE* = 0.03, |t| = 0.40).

There was no significant main effect of repetition type (β = -0.01, *SE* = 0.02, |t| = 0.41) and no significant interactions (|t|’s < 0.36) involving repetition type: Lexically repeating the head noun of the island itself vs. a phrase unrelated to the island did not affect ratings.

#### Acceptability Ratings for Lag5

**FIGURE 2:**
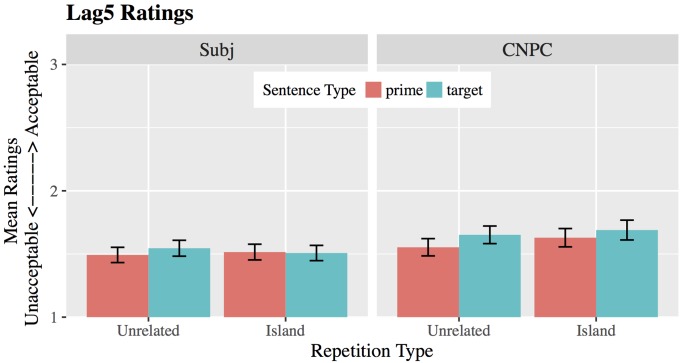
Mean ratings for CNPC and Subject islands in Lag5. Raw scores are presented on a 5-point scale, where 1 = Completely Unacceptable and 5 = Completely Acceptable. Error bars represent ±1 standard error. For visibility, we show only 1–3 points on the scale.

Ratings for prime and target sentences in Lag5 are shown in **Figure 2**. Mean ratings for CNPC islands were higher than for Subject islands, but this difference was only marginally reliable (β = 0.08, *SE* = 0.04, |t| = 1.91). Unlike in Lag1, there was no significant effect of trial type (β = 0.03, *SE* = 0.02, |t| = 1.62) and no significant sentence-by-trial interaction (β = 0.04, *SE* = 0.05, |t| = 0.91): Ratings for target sentences did not significantly differ from prime sentences, either in CNPC or Subject islands. Lag5 also showed no main or interaction effects involving repetition type (|t|’s < 1.15). Thus, in contrast to the improvements that we observed for CNPC islands in Lag1, no rating improvements were observed in Lag5, where primes and targets are separated by five intervening sentences.

### Discussion

Experiment 1 investigated acceptability rating improvements for CNPC and Subject islands in prime-target pairs. While prior work in satiation has compared rating improvements over the course of an *entire* study, our priming-style (prime-target) design allowed us to test whether factors known to affect priming might also affect satiation similarly. If so, this might provide reason to suspect that priming and satiation share underlying mechanisms. We tested two factors: (1) lexical repetition and (2) proximity of exposure between the prime and target sentences. We varied lexical repetition such that primes and targets shared either the head of the island phrase or a phrase unrelated to the island. We predicted that repetition of the head of island phrases might produce a priming ‘boost’ akin to ‘lexical boost’ effects that have been observed in priming work. In addition, we varied proximity of exposure by manipulating the number of unrelated sentences (one vs. five) between primes and targets, to probe whether potential acceptability improvements are short-term (e.g., from activation decay of structural representations) or long-term (e.g., as a result of implicit structural learning).

#### Lexical Repetition

We found no effects involving the type of lexical items repeated across prime and target sentences. The finding that acceptability ratings show no lexical repetition effects might point to a fundamental difference in the mechanisms underlying satiation and priming. However, as previously mentioned in (see *Design*), we do not compare the types of lexical repetition to a baseline condition where primes and targets do not share any lexical items. Therefore, our results do not show that there is *no* effect of lexical repetition – rather, our results provide evidence that the type of phrase that is lexically repeated does not affect the strength of priming for these sentence types. Furthermore, given that other work, including studies that examine priming in ungrammatical sentences (e.g., Kaschak and Glenberg, 2004; Luka and Barsalou, 2005; Ivanova et al., 2012a,b, 2017), found priming effects independent of ‘lexical boost’ effects, this should not be taken as evidence that priming is impossible either for CNPC or Subject islands.

#### Overall Differences in Prime-to-Target Proximity

When primes and targets were separated by only one unrelated sentence (Lag1), participants rated CNPC targets as significantly more acceptable than their primes. But, when these same island types were separated by five sentences (Lag5), we found no effect of previous exposure. In other words, acceptability ratings for CNPC islands satiated when sentences were close together, but not when they were further apart, suggesting that satiation is a short-lived effect that parallels what is predicted by lingering-activation accounts of syntactic priming (e.g., Pickering and Branigan, 1998; Branigan et al., 1999). Results from Experiment 1 therefore suggest that one factor that contributes to satiation may be a short-term priming effect that involves the lingering activation of structural representations which decay over time.^8^

#### Overall Differences between CNPC and Subject Islands

We found that CNPC islands were generally more acceptable than Subject islands. More importantly, though, we also found that CNPC islands’ acceptability ratings were *improved* by a proximate, preceding island (in Lag1), whereas Subject islands were not.

Our results provide initial evidence that satiation may be sensitive to the same factors known to affect priming. In other words, despite the indirect relationship between priming (a metric of processing ease) and acceptability ratings (a metric of well-formedness), there nevertheless appears to be a link between the two. However, our results also suggest that factors that affect priming do not seem to affect ratings across the board: They are in some way modulated by syntactic structure (e.g., CNPC island vs. Subject island). While CNPC islands were judged more acceptable in the context of a previously seen CPNC island, Subject islands did not benefit from a preceding Subject island.

#### Differences between CNPC and Subject Islands: The Stop-Being-Grammatical Task

The results of Experiment 1 suggest that rating improvements (satiation) in CNPC islands are affected by the same factors that affect priming whereas ratings for Subject islands are not. However, so far we have focused on end-of-sentence acceptability ratings, which may not reflect the processes that occur as comprehenders incrementally process CNPC and Subject islands. To gain insights into the online, incremental processing of these two islands types, we used the self-paced reading paradigm in Experiment 2. But before turning to the reading-time data, we need to address a difference between CNPC islands and Subject islands that can have implications for our interpretation of the data – namely, the relative distance between the *wh-*gap and the head of the island phrase in CNPC vs. Subject islands. Specifically, in CNPC islands (ex. 3, repeated here as 6a), the parser encounters the island-producing phrase (‘the claim’) earlier than the *wh-*gap (marked with ____) at the end of the clause. In contrast, in Subject islands (ex. 4, repeated here as 6b), the island phrase (‘a bottle of ___’) and the *wh-*gap (marked with ____) are fundamentally one and the same.

(6a) CNPC Island: Who does Mary believe [the claim] that John likes ____?
(6b) Subject Island: What does John know (that) [a bottle of ____] fell on the floor?

If it is the presence of the *gap* site – not the island-producing phrase itself – that signals “ungrammaticality”, then comprehenders may treat CNPC islands as fully grammatical until they reach the sentence-final *wh-*gap. In other words, it could be that rating improvements observed for CNPC islands – and absent for Subject islands – may not be attributable to any theoretical differences between the two islands, but simply to the fact that CNPC islands effectively *appear* grammatical for a longer amount of time.

To test this possibility, we investigate the earliest point at which comprehenders perceive CNPC islands to be ungrammatical. At the same time, this ‘stop-being-grammatical’ task also contributes to our broader goal of probing the relationship between what has been a predominantly off-line phenomena (satiation) and online facilitation effects, by proving new information about acceptability judgments at different points over the course of the sentence.

Twenty-seven native American English speakers were recruited via Amazon Mechanical Turk to participate in the stop-being-grammatical task, modeled after the stop-making-sense task (Boland et al., 1990, 1995; etc.) in Qualtrics^9^ (version 2017; Qualtrics, Provo, UT).

**FIGURE 3:**
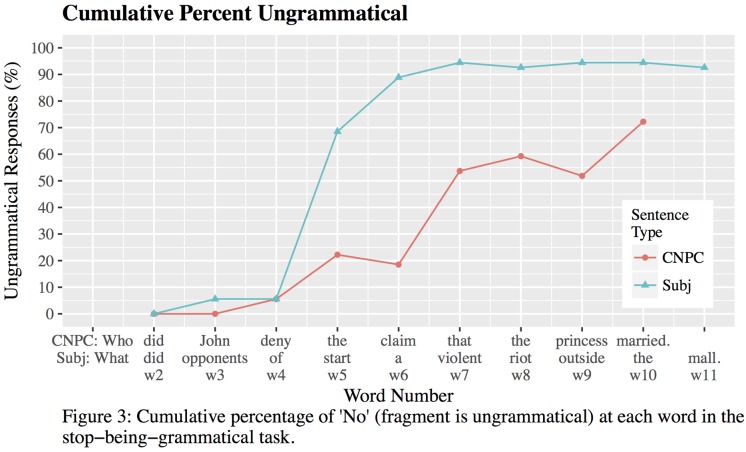
Cumulative percentage of ‘No’ (fragment is ungrammatical) at each word in the stop-being-grammatical task.

Two CNPC and two Subject islands and six filler sentences were randomly selected from Experiment 1. (Note that while Subject islands are included, they are not of interest because of the island and *wh-*gap essentially occur simultaneously. They are shown for comparison in **Figure 3**, but statistics are reported only for CNPC islands). Sentences were presented to participants in successive fragments, such that each new fragment added one more word to the end of the sentence. The initial fragment consisted of the first two words (e.g., ‘Who did,’ or ‘What did’) and subsequent fragments increased by one word. So, if participants initially saw “Who did Brandon,” the next fragment would be “Who did Brandon make”; the fragment after would contain one more word until the last word of the sentence was reached. Participants had 45 s to determine (‘Yes’/‘No’) whether each fragment could be continued to make an “acceptable”/“possible” sentence of English.

**Figure 3** shows the cumulative percentage of ‘No’ responses at each word position.^10^ At word 5 (determiner ‘the’ in CNPC islands, matrix verb in Subject islands), the number of ‘No’ responses increases for both sentence types; but at different rates for Subject vs. CNPC islands. Notably, at word 5, 70% of participants judge Subject islands to be ungrammatical with 90% of participants concurring by word 6. By contrast, although some participants judge CNPC islands to be ungrammatical at word 5, the majority do not until word 7 (complementizer ‘that’). Responses were analyzed using logistic mixed-effects regressions with random intercepts for subjects and items. We first compared responses word 4 (low rates of unacceptability) against responses at words 5 and 6 (increasing rates of unacceptability). We found a significant effect of word position for both CNPC (β = -1.88, *SE* = 0.71, |*z*| = 2.65) and Subject islands (β = -4.56, *SE* = 0.93, |*z*| = -4.92), meaning that the proportion of ‘No’ responses (i.e., ungrammatical responses) at word 4 was significantly lower than at words 5 and 6 for both island types. Contrasting words 5 and 6 yielded no significant differences for CNPC islands (β = 0.45, *SE* = 0.68, |*z*| = 0.67), but we did find a significant increase from word 5 to word 6 in Subject islands (β = -2.18, *SE* = 0.78, |*z*| = -2.79).^11^

Results from the stop-being-grammatical task suggest that judgments of (un)acceptability, like sentence processing itself, may proceed incrementally and ‘unacceptability’ is expected to begin around word 5 for both Subject and CNPC islands. More importantly, even if CNPC islands are arguably fully grammatical until the sentence-final *wh-*gap, comprehenders begin *perceiving* CNPC islands to be ungrammatical much earlier (around word 5, with a majority of comprehenders concurring by word 7). These findings argue against the potential concern that the CNPC-Subject island asymmetry in Experiment 1 was due to CNPC islands being perceived as grammatical/acceptable until the gap site at the end of the sentence. Our results suggest that comprehenders do *not* wait for the *wh-*gap to ‘decide’ whether a sentence is ungrammatical.

## Experiment 2: Self-Paced Reading

Experiment 1 provided initial evidence that acceptability ratings might be tuned to the same factors that have been found to affect online processing. However, given that prior work on satiation has mainly used acceptability ratings it is not yet known whether (i) it is end-of-sentence, meta-linguistic reflection that causes rating improvements to ‘kick in’ or whether (ii) rating improvements reflect incremental, processing facilitation. For instance, in contexts as structurally degraded as island sentences, comprehenders may rely primarily on processes outside of syntactic structure-building (e.g., plausibility, discourse context, word order, etc.). If so, rating improvements may not correspond to the type of facilitation characteristic of structural priming. Alternatively, in line with what has observed in structure-building for anomalous sentences (Ivanova et al., 2012a,b, 2017), comprehenders may nevertheless engage structural (re)integration processes even despite the type of ungrammaticality presented by island structures.

Therefore, Experiment 2 builds on Experiment 1 and the stop-being-grammatical task by directly testing whether the *online* processing of CNPC and Subject islands can be facilitated by a prior exposure. We use the self-paced reading paradigm to probe reading time slowdowns, which often stem from processing difficulty. In doing so, we probe the source of the rating improvements observed in Experiment 1, and by extension, determine whether offline rating improvements (i.e., satiation) correspond to online processing facilitation effects (i.e., priming). If recent exposure to ungrammatical structures can decrease the processing costs associated with ungrammatical structures, we might expect faster reading times for target sentences relative to their prime counterparts, which would not have the benefit of a recent facilitating exposure.

### Predictions

#### Lexical Repetition

Experiment 1 showed no effect of lexical repetition, so we do not expect differences here. We collapse repetition types in Experiment 2 to increase statistical power.^12^

#### Proximity of Exposure

Experiment 1 found that for CNPC islands, acceptability ratings improved when primes and targets were proximate (Lag1) but not when they were further apart (Lag5). This suggests that satiation may be a by-product of short-term lingering activation. If these short-term effects can be linked to those observed in short-term priming, we expect reading times to improve from primes to targets when sentences are close together (Lag1), but not when they are further apart (Lag5). But, it may also be possible that while rating improvements (satiation) are short-term, online facilitation in island sentences is the result of a more long-term priming mechanism, such as implicit learning. In the latter case, we expect prime-to-target reading time improvements regardless of whether prime and target sentences are separated by one or by five intervening sentences (Lag1 and Lag5).

In the case of Subject islands, we also expect processing difficulty to begin at word 5. However, because the parser does not postulate gaps within finite islands (Phillips, 2006), any potential processing difficulties observed here cannot be due to the filled-gap effect. In Subject islands, word 5 is the point where the parser begins to recognize the ungrammatical extraction: When the parser encounters the preposition (‘of’) at word 4, it expects that another noun phrase will follow. When it instead encounters a verb (‘start’), the parser realizes that the *wh-*filler has been extracted from within a subject phrase (i.e., a Subject island). Again, this is in line with where the majority of comprehenders in the stop-being-grammatical task (see *Differences between CNPC and Subject Islands: The Stop Being Grammatical Task*) judge Subject islands to be unacceptable.

Experiment 1 found lower ratings for Subject than for CNPC islands. Given this, one might be tempted to also predict that Subject islands might be read slower than CNPC islands. But, due to overall differences between the two sentences (e.g., word length, word frequency, etc.), we cannot compare the two sentence types directly. Rather, our comparison of interest is a sentence-by-trial *interaction*, measuring priming in CNPC vs. Subject islands that would signal this asymmetry in processing. In other words, finding that Subject and CNPC islands have different reading times (a main effect of sentence type) cannot help us to determine whether satiation and priming are linked to the same mechanisms. What is relevant is whether the same pattern of asymmetrical improvements between CNPC vs. Subject islands that was observed in Experiment 1 will also be present using in online metric. Only a sentence-by-trial interaction can speak to this asymmetry.

### Materials and Methods

#### Participants

Thirty-four (*n*_Lag1_ = 18; *n*_Lag5_ = 16) native speakers of American English from the University of Southern California participated in Experiment 2. Participants received course credit or $10 for participation. The experiment lasted roughly 45 min.

#### Procedure

The study was conducted in a quiet room at the University of Southern California. Sentences were presented using Linger (D. Rohde, MIT; Rohde, 2010).

Participants were told that sentences would start out completely masked by dashes. They were instructed to read the sentences as quickly and carefully as possible, using the ‘space bar’ to reveal each word in the sentence one-by-one. After reading the last word in the sentence, participants saw a scale ranging from 1 (Completely Unacceptable) to 7 (Completely Acceptable), where they used the mouse to rate how each sentence “intuitively” sounded to them. Participants would intermittently see a comprehension question about the sentence they just read.

#### Design

Experiment 2 used the same materials as Experiment 1. Again, two versions of the study (Lag1 vs. Lag5) were tested between-subjects. To ensure that participants were paying attention, we asked them to provide acceptability ratings for every sentence presented. However, given the extreme task differences in Experiment 1 vs. Experiment 2, we did not expect results from this rating task to be meaningful or comparable (Sag et al., 2007; Hofmeister and Sag, 2010; Hofmeister et al., 2012a,b; etc.).^13^ We report acceptability ratings for the sake of completeness, but they are not discussed further.

### Results

#### Data Analysis

Reading times below 100 ms, above 3000 ms, and more than three standard deviations above the positional mean for each condition were excluded, affecting 2 and 1.7% of the data in Lag1 and Lag5, respectively. Our region of interest began at word 5 (**Table 2**) and extended for three additional words. Because the structure of CNPC and Subject islands do not parallel each other, we do not compare them directly. Consequently, our comparison of interest is a *sentence-by-trial interaction* that compares the degree to which reading times are facilitated across island types.

Results from Lag1 and Lag5 were analyzed independently using linear mixed-effects models (Bates et al., 2015) in R (R Core Team, 2016). We included sentence type, trial type, and their interaction as fixed effects predictors. Random effect structure was determined as in Experiment 1.

#### Results from Lag1

**FIGURE 4:**
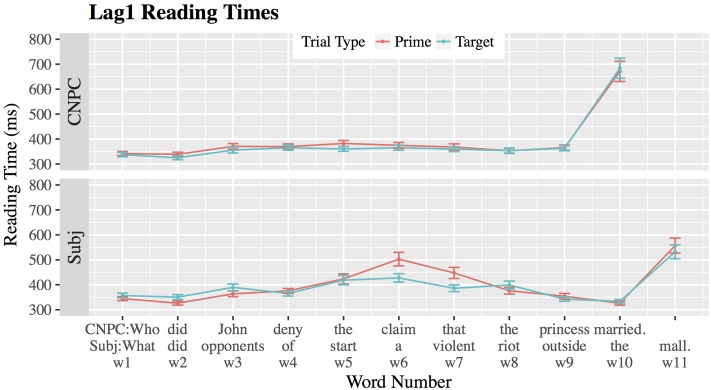
Mean reading times (ms) for CNPC and Subject islands in Lag1. Error bars represent ±1 standard error.

**Figure 4** and **Table 3** show reading times for prime and target sentences in CNPC and Subject islands in Lag1. Except at w7, we find a significant main effect of sentence type (*w5*: β = 57.04, *SE* = 22.47, |*t*| = 2.54; *w6*: β = 64.26, *SE* = 21.48, |*t*| = 2.99; *w7*: β = 27.64, *SE* = 18.31, |*t*| = 1.51; *w8*: β = 47.01, *SE* = 13.91, |*t*| = 3.38), meaning that both primes and targets for Subject islands are read slower than CPNC islands. While expected, this effect is not informative given that differences between these islands can range from individual lexical items to broader structural differences.

**Table 3:** Lag1 mean reading times for words in the region of interest.

Lag1: Reading times (ms)				

	Word 5	Word 6	Word 7	Word 8
CNPC_prime	382.44	374.73	368.03	353.16
CNPC_target	360.18	365.24	359.73	353.35
Subj_prime	423.68	502.69	447.52	376.10
Subj_target	418.54	427.61	386.02	399.23
Sig. effects detected	^∗^Sentence Type	^∗^Sentence Type	^∗^Sentence × Trial	^∗^Sentence Type
		^∗^Sentence × Trial		


*Times are shown in milliseconds.*

At word 5, CNPC islands do not show any reading time slowdowns, even though results from our stop-being-grammatical task predicted a reading time increase at this point in the sentence. Reading times for Subject islands increase at w5, consistent with results from the stop-being-grammatical task. However, we do not detect a significant main effect of trial type (β = 22.80, *SE* = 20.69, |*t*| = 1.10) or a significant sentence-by-trial interaction (β = -15.51, *SE* = 24.12, |*t*| = 0.64), meaning that reading times for primes and targets did not differ for either sentence type.

At words 6 and 7, reading times for Subject islands improve as a result of recent exposure (i.e., priming) in Subject islands (*w6*: Δ = 75.08 ms; *w7*: Δ = 61.50ms) but not for CNPC islands (*w6*: Δ = 9.49 ms; *w7*: Δ = 8.30 ms). This asymmetry in priming is corroborated by a significant sentence-by-trial interaction (*w6*: β = 59.16, *SE* = 30.40, |*t*| = 1.95; *w7*: β = 50.89, *SE* = 25.79, |*t*| = 1.97). Thus, seeing an initial Subject island facilitated processing of the subsequent Subject island. In CNPC islands, reading times for primes and targets did not differ from each other regardless of whether or not comprehenders had seen a preceding prime. Interestingly, even though a majority of participants in the stop-being-grammatical task (see *Differences between CNPC and Subject Islands: The Stop Being Grammatical Task*) rated CNPC islands as “ungrammatical” by word 7, we also find no reading time slowdown here.

After w8, reading times for Subject islands converge and appear indistinguishable. At w11, reading times increase, presumably as a result of sentence-final wrap-up effects. In CNPC islands, sentence-final wrap-up effects emerge at w10.

Recall that participants in the self-paced reading study were also asked to rate the acceptability of the sentences on a 7-point scale, to ensure they were playing attention. However, given the extreme task differences in Experiment 1 vs. 2, we did not expect these results to be meaningful (see *Design*). Analyses were conducted on *z*-scored acceptability ratings, but for ease of interpretability, we discuss raw scores.^14^ Mean ratings for CNPC island primes and targets were 2.01 and 2.11, respectively; ratings for Subject island primes and targets were 1.88 and 1.75, respectively. As expected, there were no differences between CNPC and Subject islands overall (β = -0.08, *SE* = 0.08, |*t*| = 1.06), no differences between primes vs. targets (β = 0.02, *SE* = 0.04, |*t*| = 0.46), and no sentence-by-trial interaction (β = 0.09, *SE* = 0.08, |*t*| = 1.19).

#### Results from Lag5

**FIGURE 5:**
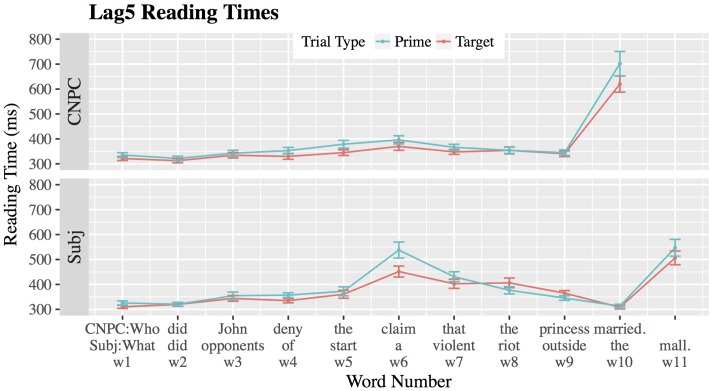
Mean reading times (ms) for CNPC and Subject islands in Lag5. Error bars represent ±1 standard error.

**Figure 5** and **Table 4** show results for reading times in Lag5. At word 5, reading times for both Subject and CNPC islands increase, but they do not differ from each other (β = 19.35, *SE* = 16.73, |*t*| = 1.16). (Recall that a main sentence type effect would not be interpretable in any case). However, we did find a significant main effect of trial type at word 5 (β = 32.65, *SE* = 16.68, |*t*| = 1.96), meaning that Subject and CNPC prime sentences were read significantly slower than their target counterparts. There was no sentence-by-trial interaction at word 5 (β = -21.44, *SE* = 23.69, |*t*| = 0.91), meaning that reading time differences between primes and targets were of the same magnitude regardless of sentence type.^15^

**Table 4:** Lag5 mean reading times for words in the region of interest.

Lag5: Reading times (ms)				

	Word 5	Word 6	Word 7	Word 8
CNPC_prime	378.66	396.12	366.34	354.13
CNPC_target	344.92	370.02	347.71	353.96
Subj_prime	372.49	537.87	431.00	376.46
Subj_target	360.43	451.67	402.53	406.34
Sig. effects detected	^∗^Trial Type	^∗^Sentence Type	^∗^Sentence Type	^∗^Sentence Type


*Times are shown in milliseconds.*

For all other words in the region of interest (w6–w8), we find only a significant effect of sentence type (*w6*: β = 81.33, *SE* = 26.39, |*t*| = 3.08; *w7*: β = 56.21, *SE* = 18.61, |*t*| = 3.02; *w8*: β = 51.63, *SE* = 16.77, |*t*| = 3.08), meaning that Subject islands were read slower than CNPC islands. However, as previously noted, this comparison is not central to the aims of Experiment 2. We also find no main effect of trial type (|*t*|’s < 1.04), meaning that the difference in prime and target reading times observed at word 5 disappeared quickly. Crucially, the sentence-by-trial interaction previously observed in Lag1 was no longer detected from w6–w8. (Despite apparent graphical differences at word 6, the sentence-by-trial interaction is not significant; it approaches marginal significance: β = 60.14, *SE* = 37.26, |*t*| = 1.614. For all other words, |*t*|’s < 1.44). At w10 and w11, reading times rise, presumably as a result of sentence-final wrap-up effects.

When we look at acceptability ratings in Lag5, we find that CNPC island ratings for primes and targets averaged 2.32 and 2.13, respectively, while Subject island ratings for primes and targets averaged 1.96 and 1.84, respectively. Unsurprisingly, CNPC and Subject islands did not differ from each other (β = -0.13, *SE* = 0.09, |*t*| = 1.44); nor did primes and targets (β = 0.07, *SE* = 0.05, |*t*| = 1.44). There was no sentence-by-trial interaction (β = -0.03, *SE* = 0.09, |*t*| = 0.29).

### Discussion

Experiment 2 used an online measure – self-paced reading times – to investigate whether the acceptability rating improvements in Experiment 1 were related to on-line island processing effects. We tested for the presence of reading time improvements, indicative of processing facilitation, for CNPC and Subject islands when primes and targets were close together (Lag1) and when they were further apart (Lag5). Based on results from Experiment 1, we predicted that if the acceptability rating improvements found in CNPC islands (but not Subject islands) reflected online processing facilitation, we should find corresponding prime-to-target reading time facilitation in CNPC islands (but not Subject islands) in Experiment 2. We also investigate whether online facilitation effects in Experiment 2 were short- or long-term. If target sentences are read faster than prime sentences in Lag1, but not in Lag5, this would point toward a short-lived priming effect. But if reading times for targets in both Lag1 and Lag5 are faster than their primes, this would suggest a long-lasting effect.

Unlike in Experiment 1, which found no rating improvements for Subject islands regardless of proximity between prime and target sentences, Experiment 2 found faster reading times for target sentences when Subject islands were separated by only one intervening sentence (Lag1). This effect lasted through several words in our region of interest. When sentences were further apart (Lag5), we found a prime-to-target facilitation localized to only one word in the region of interest. The finding that reading times for target sentences are facilitated by a preceding prime suggests that comprehenders are able to build representations of ungrammatical Subject islands and then draw on those representations to facilitate later processing of that same structure. In other words, Experiment 2 suggests that priming is possible in Subject islands. Moreover, the pattern of differences between Lag1 and Lag5 suggests that the type of priming observed for Subject islands may be attributed to rapid decay of lingering structural activation. This is similar to what has been proposed to account for short-term priming in grammatical sentences.

Conversely, reading times between prime-target pairs in CNPC islands did not appear to differ in Lag1. Despite results from the stop-being-grammatical task (see *Differences between CNPC and Subject Islands: The Stop Being Grammatical Task*), we find no reading time slowdowns associated with either the word signaling the filled-gap (w5) or the point where the processor recognizes the illicit extraction (w7) when sentences were close together. Surprisingly, we did observe a localized one-word priming effect (w5) for CNPC islands when primes and targets were far apart (i.e., in Lag5).

The reading time pattern presented by CNPC islands is difficult to interpret because no prior work has predicted a structural priming effect that *only* surfaces at longer intervals (Lag5) between prime and target. Even implicit learning accounts of priming, which predict a long-lasting effect, do not do so *in the absence* of short-term ones. Moreover, reading times for CNPC islands did not behave as one might have expected based on the stop-being-grammatical task. Results from the stop-being-grammatical task (see *Differences between CNPC and Subject Islands: The Stop Being Grammatical Task*) showed that comprehenders begin perceiving CNPC islands to be ungrammatical as early as the fifth word in the sentence (with most comprehenders concurring by the seventh word). Thus, comprehenders seem aware of the ungrammaticality of CNPC islands relatively early in the sentence. Yet, we do not detect processing difficulty (reading time slowdowns) at any point in CNPC sentences when prime and target are close together (Lag1).

It is worth noting that the reading time patterns we found for CNPC islands *do* resemble those reported for this same island type by Sag et al. (2007) and Hofmeister and Sag (2010). They investigated different issues, but used the same self-paced reading paradigm and found that reading times for CNPC islands did not differ from those in fully grammatical sentences. Crucially, their results showed that manipulating a single processing-related factor (bare *wh-*phrases vs. *which*-phrases, see *Syntactic Satiation*) was sufficient to effectively produce a reading-time ‘floor effect’ in CNPC islands. Though it may be possible that reading times for CNPC islands in Experiment 2 also exhibited a similar floor effect, this account provides little explanation for why reading times slowdowns were not detected for CNPC *primes*, which are not facilitated by prior exposure. At the moment, we leave the question of why CNPC islands did not show expected reading time slowdowns as a question for future work.

In sum, Experiment 2 leads us to conclude the following: First, reading time facilitation effects from primes to targets in Subject islands suggest that comprehenders are able to build a syntactic structure for this purportedly ungrammatical island-violation structure in real time, and that this structure can facilitate subsequent processing. Second, the results for CNPC islands suggest that structure-building for island sentences may be limited: If, following Ivanova et al. (2012a,b, 2017), we treat processing facilitation as a diagnostic for structure-building, our results indicate that comprehenders only build structures for some ungrammatical sentences. Thus, the different patterns of priming observed for Subject vs. CNPC islands reinforce the idea that the mechanisms involved in facilitating comprehension of ungrammatical sentences may not be a uniform, across-the-board phenomenon. Third, our results suggest that the proximity between prime and target sentences can affect online processing of Subject and CNPC islands, though the effect manifests itself differently for the two island types.^16^

## General Discussion

The goal of this work was to investigate the extent to which syntactic satiation (exposure-induced rating improvements in ungrammatical sentences) could be linked to syntactic priming (processing facilitation as a consequence of prior exposure). We focused on two types of island structures – Complex-NP Constraint (CNPC) and Subject islands. Our work departed from traditional approaches in satiation, where rating improvements are compared over the entire course of the study, and instead focuses on improvements between exposure-to-exposure pairs (i.e., primes vs. targets). This type of comparison allowed us to investigate whether factors known to affect online sentence processing, such as proximity of exposure and (less reliably) lexical repetition, could affect *judgments* of sentences similarly. If so, it may be possible to link priming and satiation to similar underlying mechanisms. Experiment 1 found that ratings for CNPC islands were improved by a preceding CNPC prime but only when primes and targets were separated by only one intervening sentence; when prime and target sentences were separated by five interveners, this effect was no longer detected. Subject islands, by contrast, saw no rating improvement either when prime-target pairs were close together, or when they were further apart. We further probed differences between CNPC and Subject island using the stop-being-grammatical task (see *Differences between CNPC and Subject Islands: The Stop Being Grammatical Task*). These results showed that differences between island types were not due to superficial differences in the position of the *wh-*gap (sentence-finally in CNPC vs. immediately after the head of the island phrase in Subject islands).

Given the results of Experiment 1, we then asked whether rating improvements simply reflected end-of-sentence, meta-linguistic judgment processes or whether they reflected *online* incremental comprehension processes for ungrammatical sentences. To do this, we used an online metric, reading time, to tap into structure-building and processing facilitation during the course of ungrammatical sentence comprehension. In Experiment 2, Subject islands showed reading times improvements over several words in our region of interest when primes and targets were close together (Lag1). However, when sentences were further apart (Lag5), these improvements persisted over only a single word in the region of interest. We also found that reading times for CNPC islands did not differ from each other in Lag1, suggesting that seeing one CNPC island did not facilitate CNPC processing when sentences were close together. But, when CNPC sentences were further apart, we did detect a (unexpected) single-word priming effect for CNPC islands such that target sentences were read slower than their prime counterparts.

Crucially, our results revealed a disjunction between Experiment 1 (acceptability ratings) and Experiment 2 (reading times) for both Subject and CNPC islands: Though we found no prime-to-target rating improvements for Subject islands in Experiment 1, we did find facilitated reading times in Experiment 2. This suggests that the processing of Subject islands can be facilitated (i.e., primed) by prior exposure during *online* comprehension, but that facilitation may not be sufficiently powerful to spill over to participants’ end-of-sentence offline acceptability ratings (see also Phillips, 2013 for a discussion of processing difficulty vs. well-formedness).

Meanwhile, CNPC islands did show prime-to-target rating improvements from a local exposure in Experiment 1, but those improvements did not correspond to *online* reading time/processing improvements in Experiment 2. The lack of reading-time priming effects in CNPC islands may suggest that comprehenders do not construct a *syntactic* representation for CNPC islands in real time. Instead, we suggest that the acceptability rating improvements we observed with CNPC islands may be attributable not to *structural* priming, but to a different type of adaptation by the processor. For example, prior work on the processing of ungrammatical sentences has shown that there are many non-syntactic alternatives – based on frequency (e.g., Hare et al., 2003), discourse context (e.g., Spivey-Knowlton et al., 1993), plausibility (e.g., Ferreira, 2003), and simple word order heuristics (e.g., Ferreira, 2003) – through which comprehenders might choose to interpret an anomalous structure (see Pickering and van Gompel, 2006 for review). If alternative routes are more accessible than the syntactic structure-building route when comprehenders encounter a CNPC island, they will presumably opt for a non-syntactic approach. Thus, our failure to detect online facilitation effects with CNPC islands may be related to the viability of a non-structural processing route. Further research is needed to investigate this more directly. Under this view, the reading time slowdowns that we detected in the Lag5 group for CNPC islands hint that facilitation effects – even when not *structurally* driven – may be sensitive to the distance between exposures.

Taken together, our work points to some links between satiation (improvements in acceptability) and priming (facilitation in processing). First, we find that priming – and by extension, structure building – may be possible in Subject islands. And, while online processing effects were not reflected in end-of-sentence rating improvements, the presence of an online facilitation effect suggests that we cannot rule out the possibility of priming in ungrammatical sentences. Further, improvements observed for Subject and CNPC islands appear to be sensitive to the distance between prime and target sentences. Specifically, improvements – in terms of ratings (Experiment 1) or reading times (Experiment 2) – that emerged as a result of prior exposure were present when sentences are close together (Lag1), but absent when exposures are further apart (Lag5). One possibility, then, may be that both satiation and priming are linked to a short-term mechanism such as residual activation of structural representations that decay rapidly. Importantly, our results do not suggest that satiation should simply be equated with priming. While some of the results here may be compatible with ‘satiation as priming,’ it is premature at this stage to equate the two without further investigating factors such as the role of lexical repetition, (the absence of) long-term priming effects, etc.

### Implications for Theories of Island Constraints

Prior work has sought to directly address which factors might contribute to the different patterns of satiation across island types (cf. Hiramatsu, 2000; Kluender, 2004; Sag et al., 2007; Crawford, 2012; Chaves and Dery, 2014; *inter alia*). That issue is not the main focus of the experiments reported in this paper. However, both Experiments 1 and 2 suggest that Subject islands and CNPC islands behave differently. Therefore, it may be reasonable to suggest that what has been grouped under the same ‘satiation’ umbrella may actually be two different underlying mechanisms, targeting different kinds of island violations, that happen to yield superficially similar consequences.

Prior work has attempted to classify island constraints under different syntactic (e.g., Ross, 1967; Huang, 1982; Chomsky, 1986; Rizzi, 1990) or semantic (e.g., Szabolcsi and Zwarts, 1993) mechanisms. To date, though, these typologies (e.g., “strong” vs. “weak” island effects) are neither very straight-forward nor fully agreed-upon (Szabolcsi and den Dikken, 2003; Szabolcsi, 2006; etc.). However, theories (Ross, 1967; Kluender, 1998, 2004; Hiramatsu, 2000; etc.) that suggest a typological distinction between CNPC and Subject islands may be able to capture the pattern of results presented here. For instance, some accounts consider CNPC islands to be “weak” and Subject islands to be “strong” by virtue of the severity of the violation (quantified in terms of subjacency violations).^17^ Though our work cannot speak to the validity of these classifications, it is worth noting that our results do provide evidence against grouping CNPC and Subject islands as a natural class. Clearly, further work is required to pinpoint what precisely defines the asymmetric satiation and priming effects that we observe.

The different pattern of behaviors for CNPC and Subject islands may also speak to an ongoing debate concerning the status of island violations in general. On one hand, with CNPC islands we (unexpectedly) found reading time differences between primes and targets when primes and targets were far apart but not when they were close together. This could be argued to lend support to accounts that primarily attribute island effects to processing effects (e.g., Kluender and Kutas, 1993; Kluender, 1998, 2004; Sag et al., 2007; Hofmeister and Sag, 2010; Pearl and Sprouse, 2012, 2015; but see Phillips, 2013). On the other hand, online facilitation effects for Subject islands were not strong enough to ‘spill over’ to acceptability improvements. This suggests that while the acceptability of island sentences may be affected by processing-related factors, attempts to locate island effects wholly outside the grammar are insufficient (Ross, 1967; Chomsky, 1986; Sprouse et al., 2012a,b; Phillips, 2013; Yoshida et al., 2014). As in the case of satiation, it may be that the role of processing-related factors may affect these two island types differently.

### Implications for Methodology

Traditional measures of satiation have relied on acceptability judgments, which is a consequence of how satiation as a phenomenon has been defined. However, our results show that there is a benefit to looking at satiation using multiple methods. Ratings from the acceptability judgment task (Experiment 1) provide a ‘first look’ into the potential link between satiation and priming. Strikingly, once we adapted the task to an online measure (Experiment 2), it became apparent that acceptability ratings alone did not allow us to fully differentiate between the mechanisms targeting the two different sentence types. The emerging picture is admittedly complex, but adds new empirical evidence to a subfield of linguistics – satiation research – that has been characterized by a lack of consensus from the outset.

Finally, while prime-target proximity effects have been thoroughly investigated in the priming literature, our work is the first (to our knowledge) to take some initial steps toward investigating proximity in studies of acceptability ratings. Therefore, an independent contribution of our work is to highlight the need to control for distance between targets in acceptability judgment tasks.

## Ethics Statement

All studies reported in this paper were reviewed and approved by the University of Southern California University Park Institutional Review Board, which is fully accredited by the Association for the Accreditation of Human Research Protection Programs (AAHRPP). Due to the nature of the experiments, the Institutional Review Board determined that written consent was not needed.

## Author Contributions

MD and EK conceptualized and designed the experiments. MD acquired the data and conducted the statistical analyses. Both MD and EK interpreted the data and wrote the manuscript.

## Conflict of Interest Statement

The authors declare that the research was conducted in the absence of any commercial or financial relationships that could be construed as a potential conflict of interest.

## Footnotes

1 Snyder (2000) tested seven different structures, finding satiation for *whether*-islands as well. But, because they do not allow us to incorporate repetition type as a factor, we exclude them from the current study. Snyder did not find satiation in want-for, that-trace, adjunct islands, or left branch sentences.

2 Ross (1967) distinguishes between two sub-categories of CNPC violations: extraction out of a relative-clause NP and extraction out of a sentential complement NP. Following Snyder (2000) and others, we focus on only sentential complements.

3 While numerous production-based studies found priming even in the absence of lexical repetition, most comprehension-based studies found priming *only* when the prime and target have lexical overlap. An open question is whether this difference stems from the different tasks used to study priming in the two modalities or whether priming mechanisms in production and comprehension are fundamentally distinct (see Tooley and Traxler, 2010 for review).

4 Ivanova et al. (2012a, 2017) also found that there was a priming ‘boost’ when verbs were the same. However, because priming was observed even when there was no lexical overlap, they concluded that even priming of anomalous sentences was lexically independent (but see Ivanova et al., 2012b).

5 We also excluded 11 participants who were either inattentive or did not understand the task. These participants responded to more than one comprehension questions incorrectly, rated grammatical fillers as ‘Completely Unacceptable,’ and/or were very slow in completing the experiment.

6 http://www.qualtrics.com

7 Random effects started out fully crossed and fully specified; they were reduced (starting with by-item effects) via model comparison, wherein only random effects that contributed significantly to the model (*p* > 0.05) were included (Baayen et al., 2008).

8 Even though we discuss numerical differences between Lag1 and Lag5, between-group effects were not compared directly. Because our study is the first of its kind to explore the links between satiation and priming in this way, while also comparing different island types, it was not designed with the statistical power to detect a 3-way, between-subjects interaction. Between-subjects effects are difficult to detect, especially without sufficient statistical power. The situation is further complicated by the well-known observation that structural priming effects are relatively small, and the fact that the effect of interest is a three-way interaction between sentence type, trial type and lag. Even though our sample size is in line with current psycholinguistic work, we do not expect to be able to detect this kind of interaction. Additional exploratory analyses suggest that doing so would require a sample size much larger than what is standard in psycholinguistics. Nevertheless, we acknowledge that further work requires a more vigorous focus on effect sizes, power, and sufficient sample size.

9 http://www.qualtrics.com

10 The cumulative percentage of responses at any position is fundamentally dependent on the number of ‘No’ responses prior to that position. To minimize this dependence, we also calculate adjusted percentages such that the number of ‘No’ responses was out of the total of “remaining possible no” at each position (Boland et al., 1990). Adjusted percentages showed the same pattern.

11 While a the statistically significant difference between words 5 and 6 in Subject islands is interesting, it is ultimately irrelevant to the central aims of the stop-being-grammatical task. Namely, to determine the *first* point at which the sentence becomes ungrammatical. Therefore, we do not discuss reasons for difference between words 5 and 6 in Subject islands.

12 At the request of a reviewer, additional analyses were carried out with repetition type as a fully crossed fixed effect. It was also included in the by-subject and by-item random effect structures. Model reduction was done as previously described. In both Lag1 and Lag5, there were no significant main or interaction effects involving repetition type during any portion of our region of interest.

13 Participants in Experiment 1 were shown the full sentences during the rating task. Participants in Experiment 2, however, first read the sentence word-by-word and then rated the sentence from memory after the sentence had disappeared from the screen.

14 In all cases, analyses performed over raw score ratings showed the same pattern as *z*-scored ratings.

15 At the request of an reviewer, we performed separate analyses for CNPC and Subject islands at w5 with the hunch that prior analyses were insufficiently powered to detect the interaction effect. This subsequent analysis showed that main effect of trial type was primarily driven by CNPC islands. However, this does not impact the main claims of this paper. We provide additional discussion of the localized one-word priming effect for CNPC islands in Section “Discussion.” The lack of an effect for Subject islands at w5 is consistent with our claims that priming does not occur when primes and targets are further apart.

16 Again, numerical differences between Lag1 and Lag5 were not compared directly, as discussed in footnote 7.

17 In other accounts, both CNPC and Subject islands are considered “strong” islands; but, these accounts cannot explain the difference between island types observed here. We, therefore, use the terminology “weak” and “strong” here simply to follow the convention that was used by the relevant work (Kluender, 1998, 2004; Hiramatsu, 2000; etc.). Though, as noted above, the distinction between “weak” and “strong” islands is not straightforward and still an open question (Szabolcsi and den Dikken, 2003; Szabolcsi, 2006; etc.). What is critically relevant is that – regardless of terminology – prior work which has independently suggested a distinction between CNPC and Subject islands has the potential to account for differences observed here.
